# IL-6/STAT3–Mediated miR-181a-5p in Bone Marrow–Derived Mesenchymal Stem Cells Regulates Th17/Treg Balance in Experimental Periodontitis

**DOI:** 10.1016/j.identj.2026.109675

**Published:** 2026-06-11

**Authors:** Si Wu, Jie Wang, Deqin Yang

**Affiliations:** a The Affiliated Stomatological Hospital of Chongqing Medical University, Chongqing Key Laboratory of Oral Diseases, Chongqing Municipal Key Laboratory of Oral Biomedical Engineering of Higher Education, Chongqing Municipal Health Commission Key Laboratory of Oral Biomedical Engineering, Chongqing, China; b Department of Conservative Dentistry and Endodontics, Shanghai Stomatological Hospital and School of Stomatology, Shanghai Key Laboratory of Craniomaxillofacial Development and Diseases, Fudan University, Shanghai, China

**Keywords:** Periodontitis, MicroRNA-181a-5p, IL-6, BMMSCs

## Abstract

**Objective:**

Periodontitis adversely affects oral and overall health. Bone marrow–derived mesenchymal stem cells (BMMSCs) play regulatory roles in the immune system, and the exploration of therapeutic strategies involving BMMSCs has garnered significant attention in the context of inflammatory pathological processes. Interleukin-6 can regulate microRNA expression by activating the signal transducer and activator of transcription 3 (STAT3) and functions in some systemic diseases. Notably, the regulatory mechanisms between IL-6 and miR-181a-5p in BMMSCs underlying periodontitis are still unknown. This study aims to uncover the regulatory mechanisms between IL-6 and miR-181a-5p and create a new treatment strategy for periodontal tissue regeneration.

**Methods:**

In this study, Western blot was used to assess IL-6 and STAT3 levels. Quantitative real-time polymerase chain reaction assessed the expression of miR-181a-5p and the relative genes. Enzyme-linked immunosorbent assay detected the content of IL-6. The Cell Counting Kit-8 was used to evaluate cell viability. Alkaline phosphatase staining was used to analyse the osteogenesis of cells. Chromatin immunoprecipitation and dual luciferase activity assays were used to verify the binding relationship between miR-181a-5p, STAT3, and IL-6. Flow cytometry was used for identifying cell types. Kyoto Encyclopedia of Genes and Genomes (KEGG) and Gene Ontology analysed potential signal pathways of genes targeted by miR-181a-5p. In addition, the present research was further investigated using a ligature-induced periodontitis model in mice. The micro–computed tomography, haematoxylin and eosin, and tartrate-resistant acid phosphatase (TRAP) assays were used for histologic analysis of the degree of inflammation and bone recovery in the experimental periodontitis and treatment group.

**Results:**

IL-6 and miR-181a-5p were upregulated in experimental periodontitis in mice. The IL-6 recombinant protein promoted mBMMSC proliferation and upregulated miR-181a-5p levels via STAT3. Functional inhibition of miR-181a-5p alleviated the T helper 17 (Th17) cell/regulatory T cell (Treg) immune imbalance triggered by IL-6 stimulation. Injection of miR-181a-5p inhibitor preconditioned mBMMSCs significantly promoted periodontal tissue regeneration, and osteoclasts and bone resorption were reduced significantly.

**Conclusion:**

IL-6 may contribute to the progression of experimental periodontitis in mice via modulating STAT3‑mediated miR‑181a‑5p expression in mBMMSCs and may potentially influence periodontal tissue regeneration through regulating the Th17/Treg balance.

## Introduction

Periodontitis is a chronic inflammatory oral disease associated with a persistent local immune response disorder in the periodontal microenvironment.^1^ The progression of periodontitis can induce severe destruction of periodontal tissues, especially alveolar bone resorption. Persistent progressive periodontitis may ultimately lead to tooth loss, adversely affecting chewing efficiency and threatening overall health.^2^ Research has characterised periodontitis as an infectious disease induced by immune dysregulation and driven by periodontal pathogenic microorganisms and their virulence factors. In gingival tissues, chronic pathogenic stimulation activates immune cells and leads to excessive inflammatory activation.^3^ The immune dysregulation disrupts physiological immune homeostasis, inducing irreversible pathologic damage to periodontal soft and hard tissues. Accumulating evidence confirms that in the periodontal microenvironment, an excessive inflammatory response disrupts the physiological balance between injury and repair, thereby promoting progressive degradation of periodontal soft tissues and alveolar bone. Therefore, uncovering the mechanisms of immune disorders in the periodontitis microenvironment is fundamental for exploring targeted immunomodulatory strategies for periodontal disease intervention.

Recently, the imbalance between T helper 17 (Th17)/regulatory T cells (Tregs) has been found to be a crucial component of periodontitis pathogenesis.^4^^,^^5^ Th17 cells belong to the CD4^+^ T-cell line and play a significant role in aggravating inflammation. Not only do they participate in combating stimuli from microorganisms, but they can also activate inflammation and bone resorption by recruiting neutrophils and producing IL-6, IL-23, and IL-17. Research showed that antibodies targeting IL-17 can protect mice from severe periodontitis.^6^ Tregs express forkhead box protein 3 (FOXP3) specifically and regulate immune reactions by releasing immunosuppressive cytokines.^4^ The complex relationships and imbalance between Th17 cells and Tregs may be important factors in the development of periodontitis. The balance of regulatory agents in Th17/Tregs, such as IL-35 and calcitriol, has been shown to have beneficial effects in periodontitis therapy. The relationships underlying the RANKL/RANK/OPG pathway and the Th17/Treg imbalance are also considered key mediators connecting periodontal bone metabolism and the immune system.^7^^,^^8^ Thus, elucidating the regulatory mechanisms of Th17/Treg balance provides a basis for exploring potential immunomodulatory therapeutic strategies for periodontal tissue regeneration in periodontitis.

As the precursor cells of osteoblasts, bone marrow–derived mesenchymal stem cells (BMMSCs) have emerged as promising candidates for regenerative therapy of periodontitis owing to their regulatory effects on immune cells of T lymphocytes, B lymphocytes, and neutrophils by inhibiting their differentiation and proliferation.^9^^,^^10^ Accumulating evidence has confirmed that BMMSCs can suppress excessive inflammatory responses and interact with immune cells through paracrine and intercellular crosstalk.^11^^,^^12^ For periodontal tissue regeneration, BMMSCs affect the differentiation balance of immune cells and reconstruct local immune homeostasis. Furthermore, BMMSCs can alleviate chronic inflammatory injury by inhibiting proinflammatory Th17 differentiation and promoting anti-inflammatory Treg polarisation.^13^^,^^14^ Nevertheless, the potential mechanisms underlying BMMSCs that participate in immune regulation in periodontitis, particularly the noncoding RNA regulatory network, still need further study. Exploring preconditioning strategies is helpful to enhance the immunomodulatory capacity of BMMSCs, which is essential for promoting clinical translation of stem cell therapy in periodontitis. In recent years, mesenchymal stem cell–derived extracellular vesicles have been regarded as potential candidates for cell-free regenerative therapy, which exert a satisfactory therapeutic performance in the immunomodulatory treatment of periodontitis.^15^^,^^16^

The expression of IL-6 is upregulated in serum and gingival crevicular fluid of patients with periodontitis.^17^, ^18^, ^19^ As a systemic marker of immune activation and inflammation, IL-6 assists the host in initiating defence mechanisms by activating the acute phase of the immune response, but IL-6 disorders may cause pathologic impacts in chronic diseases mediated by the immune system and excessive inflammatory response. In periodontitis, Th17 cells are enriched and activated in periodontal tissues, and IL-6 helps regulate Th17 cell differentiation. IL-6 can bind to the IL-6 receptor, activating the JAK family through phosphorylation of gp130, thereby promoting the differentiation of Th17 cells. Notably, IL-6 can also function in some diseases via inducing the transcription and expression of some microRNAs by activating the STAT3 signal.^20^

MiR-181a-5p belongs to the miR-181 family, which is not only enriched in lymphatic tissues but also has a significant impact on the development and age-related defects of thymic T cells.^21^^,^^22^ Our previous studies suggested that miR-181a-5p influences CD4^+^ T lymphocyte viability by regulating the functions of mBMMSCs. MiR-181a-5p in mouse BMMSCs (mBMMSCs) of osteoporosis is upregulated and influences the CD4^+^ T lymphocyte apoptosis, thereby affecting bone remodelling.^23^ Underlying the pathogenesis of periodontitis, the effects of single cytokines on certain lymphocyte subsets have been well studied. However, the mechanisms of miR-181a-5p that underlie the regulation of Th17/Treg balance and cell crosstalk in periodontitis still require further study.^23^ The present research aims to study the role of IL-6/miR-181a-5p on Th17/Treg balance in BMMSCs of periodontitis in vivo and in a mouse periodontitis model, furthermore exploring the effects of this potential regulatory mechanism on periodontal tissue regeneration.

## Materials and Methods

### Animals

Eight-week-old C57BL/6J mice (male) were purchased and housed in the Laboratory Animal Center of Chongqing Medical University under specific pathogen–free conditions. The animal experiments were performed strictly in accordance with the guidelines approved by the Animal Ethics Committee of Chongqing Medical University, with Approval Number 2024 (071). All animal experiments in this study were performed in accordance with the ARRIVE (Animal Research: Reporting of In Vivo Experiments) guidelines. Experimental periodontitis was established in the maxillary second molar of mice using the silk ligation method.^18^ Briefly, suture thread (5-0) was placed around the cervical region of the molar using ophthalmic forceps, and the ligature was tightly knotted on the palatal side. After 2 weeks of ligation, mice were euthanised, and the maxillary tissues were collected for subsequent detection. For in vivo therapeutic intervention, mice were randomly divided into different groups (n = 5 per group): normal group, periodontitis group, and mBMMSC treatment groups, including the IL-6 group (mBMMSCs pretreated with IL-6), *Stat3*–small interfering RNA (siRNA) group (mBMMSCs pretreated with *Stat3*-siRNA), miR-181a-5p inhibitor NC group (mBMMSCs pretreated with inhibitor NC), and miR-181a-5p inhibitor group (mBMMSCs pretreated with miR-181a-5p inhibitor). In this study, biological replicates refer to independent samples from different mice. After the silk ligation was removed, preconditioned mBMMSCs were injected into the secondary molar. For cell injection, 2 injection sites were placed on both the buccal and lingual sides with 5 × 10^4^ cells per site twice a week. Two weeks after treatment, the maxillary tissues were collected for subsequent research.

### Cell culture

mBMMSCs and CD4^+^ T lymphocytes were isolated and cultured as previously described.^23^ For mBMMSC collection, mice were euthanised, and long bones were separated and washed with sterile phosphate-buffered saline (PBS) solution (Biosharp) containing 10% penicillin streptomycin (Hyclone). After cutting the metaphysis of long bones, bone marrow was flushed out from the marrow cavity using α-MEM (Gibco) culture medium with 10% foetal bovine serum (Lonsera). For CD4^+^ T lymphocytes, mice were euthanised, and spleens without peripheral connective tissues were separated and kept in precooled PBS solution. Under sterile conditions, the spleen was placed in RPMI 1640 medium (Gibco), and a 100-μm cell strainer was used to crush the spleen for cell suspension. Supernatant was removed after centrifugation at 4 °C. The red blood cells were totally dissolved by the lysis buffer (Biosharp). The cells were cultured in special medium (RPMI 1640) and culture dishes that were pretreated with 5 μg/mL CD3 and CD28 antibodies overnight at 4 °C. The 0.4-μm transwell coculture system (Corning) was used for culturing both mBMMSCs and T lymphocytes.

The transfection was performed when cell growth reached 70% to 80%. Opti-MEM (Gibco) was used to dilute Lipofectamine 3000 (Invitrogen), miR-181a-5p mimic/inhibitor (RiboBio), mimic/inhibitor NC (RiboBio), *Stat3*-siRNA (Tsingke), and siRNA-NC (Tsingke) for 3 minutes at room temperature. Then, Lipofectamine 3000 was mixed with the other dilutions and incubated for 15 minutes. The final concentration was adjusted to 50 nM with culture medium. After 48 hours of transfection, the mBMMSCs were used in subsequent experiments. As mBMMSC growth reached 80%, the cells were incubated with 0 μM, 1 μM, and 2 μM STAT3 inhibitor (WP1066, Beyotime) for 24 hours to suppress STAT3 function.

### Crystal violet staining

mBMMSCs were cultured in a 6-well plate for 1000 cells/well. After 14 days of culturing, cells were fixed with 4% paraformaldehyde (Biosharp) and stained with crystal violet (Solarbio) for 20 minutes. The PBS solution was used to wash the cells 3 times, and the plates were dried at room temperature. A microscope (Nikon) was used for observation and taking photos.

### Cell Counting Kit-8 analysis

For the mBMMSC culture, 96-well plates were used, with 1000 cells/well. After being cultured for 1, 3, 5, or 7 days or stimulated by various concentrations (0.1, 1, 10, 25, 50, and 100 ng/mL) of mouse IL-6 recombinant protein solutions (KeyGEN) for 24 hours. mBMMSCs were incubated in Cell Counting Kit-8 (MedChemExpress) solution at 37 °C for 2 hours. OD values for each well were measured using a microplate reader (Thermo Fisher Scientific) at 450 nm.

### Osteogenesis and alkaline phosphatase staining

mBMMSCs were seeded into 6-well plates for osteogenic induction and alkaline phosphatase staining. As cell growth reached 80%, the osteogenic induction medium (Cyagen) was used to induce osteogenic differentiation of mBMMSCs. The alkaline phosphatase activity of the cells was detected by the Assay Kit (Beyotime). A microscope (Nikon) was used for observation.

### Flow cytometry analysis

As described in the manufacturer’s protocol, the ratios of Tregs (CD4^+^, CD25^+^, FoxP3^+^) and Th17 cells (CD4^+^, IL-17^+^) among total CD4^+^ T cells were measured by flow cytometry. A cell suspension of 1 × 10^6^ cells/500 μL was prepared in PBS buffer containing 3% foetal bovine serum and 5 μL of antibodies. After incubating at room temperature in the dark for 30 minutes, the supernatant was removed by centrifuging. PBS buffer-washed cells were washed twice, and 500 μL of cell suspension was prepared for flow cytometry. A flow cytometer (BD Influx) and FlowJo software v10.2 (BD) were used for testing and analysis, respectively. The antibodies (antimouse) used were as follows: CD90-APC, CD105-PE, CD31-PE-Cy7, CD45-FITC, FITC-CD4, and APC-IL17A (1:100; Biolegend). The mBMMSCs were pretreated by foxp3/transcription factor immobilisation/membrane lysis reagent (Elabsciense) before the incubation of antibody PE-antimouse FOXP3 (1:100; Elabsciense).

### Enzyme-linked immunosorbent assay

After centrifugation for 20 minutes, the supernatant of mouse peripheral blood was obtained, and the content of IL-6 was measured using an enzyme-linked immunosorbent assay (ELISA) kit (JINGMEI BIOTECHNOLOGY). OD values for each well were read at a 450-nm wavelength. A standard linear regression curve was used to calculate the concentrations of the serum samples.

### Quantitative real-time polymerase chain reaction

Trizol (Invitrogen) was used for obtaining total RNA. Notably, microRNA extraction had to be transferred at –80 °C overnight after adding isopropanol. HiScript III RT SuperMix and ChamQ Universal SYBR qPCR Master Mix (Vazyme) were used to detect the expression of relative genes. The polymerase chain reaction (PCR) primer (Tsingke) sequences are shown in Table 1. The microRNA reverse transcription and quantitative real-time PCR (RT-qPCR) were performed using a kit from RiboBio. MiR-181a-5p and U6 sequences are shown in Table 2.

**Table 1:** Primer sequences.

Primer	Sequence
*Gapdh*	Forward: TGTGTCCGTCGTGGATCTGA
	Reverse: TTGCTGTTGAAGTCGCAGGAG
*Il-1β*	Forward: TCCAGGATGAGGACATGAGCAC
	Reverse: GAACGTCACACACCAGCAGGTTA
*Tnf-α*	Forward: GCCTCTTCTCATTCCTGCTT
	Reverse: TGGGAACTTCTCATCCCTTTG
*Il-6*	Forward: GGGACTGATGCTGGTGACAA
	Reverse: ACAGGTCTGTTGGGAGTGGT
*Stat3*	Forward: GGAGCCCACCAAGAACGAT
*Ifit3*	Reverse: GTCACCAGCATCAGTCCCAA Forward:GCTCAGGCTTACGTTGACAAGG Reverse: CTTTAGGCGTGTCCATCCTTCC

**Table 2:** Primer sequences of miR-181a-5p and U6.

Primer	Sequence
U6	Forward: CTCGCTTCGGCAGCACA
	Reverse: ACGCTTCACGAATTTGCGT
miR-181a-5p	Forward: AACAUUCAACGCUGUCGGUGAGU
	Reverse: UCACCGACAGCGUUGAAUGUUUU

### Western blot

mBMMSCs were cultured in 6-well plates for total protein extraction. Total proteins were extracted using a kit (Beyotime). After a 30-minute ice bath, the cell lysis mixture was centrifuged at 4 °C, and the supernatant was diluted with loading buffer and boiled to denature. The 10% gels were prepared using a kit (Epizyme Biotech) and used for sodium dodecyl sulphate–polyacrylamide gel electrophoresis. Protein signals were detected by the BeyoECL Plus kit (Beyotime) and the Chemi DOCTMXRS^+^ Imaging system (BIO-RAD). Antibodies used were β-actin (1:1000, ab170325; Abcam), IL-6 (1:1000, sc-32296; Santa Cruz), STAT3/p-STAT3 (1:1000, AF6294/AF3293; Affinity), goat-anti-rabbit IgG horseradish peroxidase secondary antibodies (1:5000, ab205718; Abcam), and goat-anti-mouse IgG horseradish peroxidase secondary antibodies (1:5000, ab205719; Abcam).

### Dual‑luciferase reporter assay

The JASPAR database was used to predict STAT3 binding sites on promoter regions of miR-181a. The synthetic mutation fragments of predicted site 1 (miR-181a WT 1, miR-181a MUT 1) and site 2 (miR-181a WT 2, miR-181a MUT 2) were cloned into pGL3 and then cotransfected with STAT3. The cell suspension was collected, and luciferase activity was detected by a kit (YEASEN) according to the protocol.

### Chromatin immunoprecipitation

mBMMSCs were treated with IL-6 (50 ng/mL) for 24 hours, and the untreated group served as the negative control. Cells were cross-linked with formaldehyde for chromatin immunoprecipitation (ChIP). Cell nuclei and chromatin were prepared using the ChIP kit (Bes5001; BersinBio) and the ultrasonic lysis method. The antibodies used were STAT3 (1:100, 9139s; CST) and rabbit-IgG antibody (1:100, 3000-0-AP; Proteintech). RT-qPCR was performed to quantify the DNA samples. Primer sequences are shown in Table 3.

**Table 3:** Primer sequences for chromatin immunoprecipitation.

Primer	Sequence
*miR-181a-5p-site1*	Forward: GTACTGGCTGCTTTTCCAGA
	Reverse: TTTATGTGTATGGGTGTTTGCTT
*miR-181a-5p-site2*	Forward: ATCCTGCAGCTCATTTCTGC
	Reverse: TCTTAAGGCTGGCTTGGTTG

### Cell transcriptome RNA sequencing

mBMMSCs were seeded in 10-cm plates, and the transfections of miR-181a-5p mimic/mimic NC were performed when cell growth reached 80%, with 3 replicates for each group. Samples were processed by Majorbio Bio-pharm Biotechnology. The RNA sequencing (RNA-seq) transcriptome library for the samples was generated from 1 μg total RNA using the Illumina NovaSeq Reagent Kit to build a transcriptome database. DESeq2, limma, and edgeR software were used for data analysis and forming the transcriptome library, with the criteria being |log_2_ fold change| ≥ 1 and *P* < .05. Relative cellular pathways and functional networks of differentially expressed genes were analysed by the Gene Ontology database (http://geneontology.org), Kyoto Encyclopedia of Genes and Genomes (https://www.genome.jp/kegg), and STRING database (http://string-db.org).

### Micro–computed tomography analysis

Micro–computed tomography (CT) assay was performed on the mouse maxillae before decalcification. The parameters of micro-CT (vivaCT 40) were set at 15 μm, 70 kVp, and 110μA. Bone volume/total volume (BV/TV) and the cementoenamel junction–alveolar bone crest (CEJ-ABC) distances were analysed by CT Analyzer software.

### Histologic analysis

Mice maxillae were decalcified in 10% disodium EDTA for 4 weeks and embedded in paraffin after dehydration. A haematoxylin and eosin staining kit (Solarbio) and tartrate-resistant acid phosphatase kit (Servicebio) were used for bone formation analysis. A slide scanning system (VS200; OLYMPUS) was used to observe sections.

### Statistical analysis

Data were analysed by the 2^–ΔΔ^CT method in GraphPad Prism 10.1 (GraphPad Software). The relative messenger RNA expression was normalised to GAPDH. MiR-181a-5p expression was normalised to U6. All data are presented as mean ± standard deviation. Prior to statistical analysis, the Shapiro-Wilk test was used to assess normality, and Levene’s test was performed to evaluate homogeneity of variance across all datasets. Only data with a normal distribution and homogeneous variance were included for further comparison. The 95% confidence intervals were calculated to estimate the reliability of intergroup differences. In animal experiments, each group contained 5 biological replicates. All in vitro cell assays were performed in triplicate. Statistical comparisons were performed using the Student *t* test or 1-way analysis of variance, with *, **, ***, and **** denoting *P* values less than .05, .01, .001, and .0001, respectively. *P* < .05 was considered statistically significant.

## Results

### IL-6 and miR-181a-5p were upregulated in periodontitis

Periodontitis was established in the maxillary second molar of mice using the silk ligation method, while mice in the control group had no treatment (Figure S1A). After 2 weeks of silk ligation, micro-CT and histologic analysis showed that both hard and soft periodontal tissues of mice in the control group were normal, with no obvious alveolar bone resorption (Figure S1B). The periodontitis mice showed a certain extent of alveolar bone resorption, as the ratio of BV/TV was significantly lower than that of the control group. Moreover, the CEJ-ABC distances of the buccal and palate sides markedly increased (Figure S1C). For haematoxylin and eosin staining, more inflammatory cell infiltration was observed in the maxillary tissues of periodontitis mice compared with the control group. Furthermore, the alveolar bone crest of the secondary molar was obviously absorbed, and collagen fibres in the periodontal ligament were disordered. Combined with root migration of the epithelium, the disappearance of the alveolar ridge crest was replaced by fibres, and the lamina propria was infiltrated by inflammatory cells (Figure S1D).

**Fig. 1:**
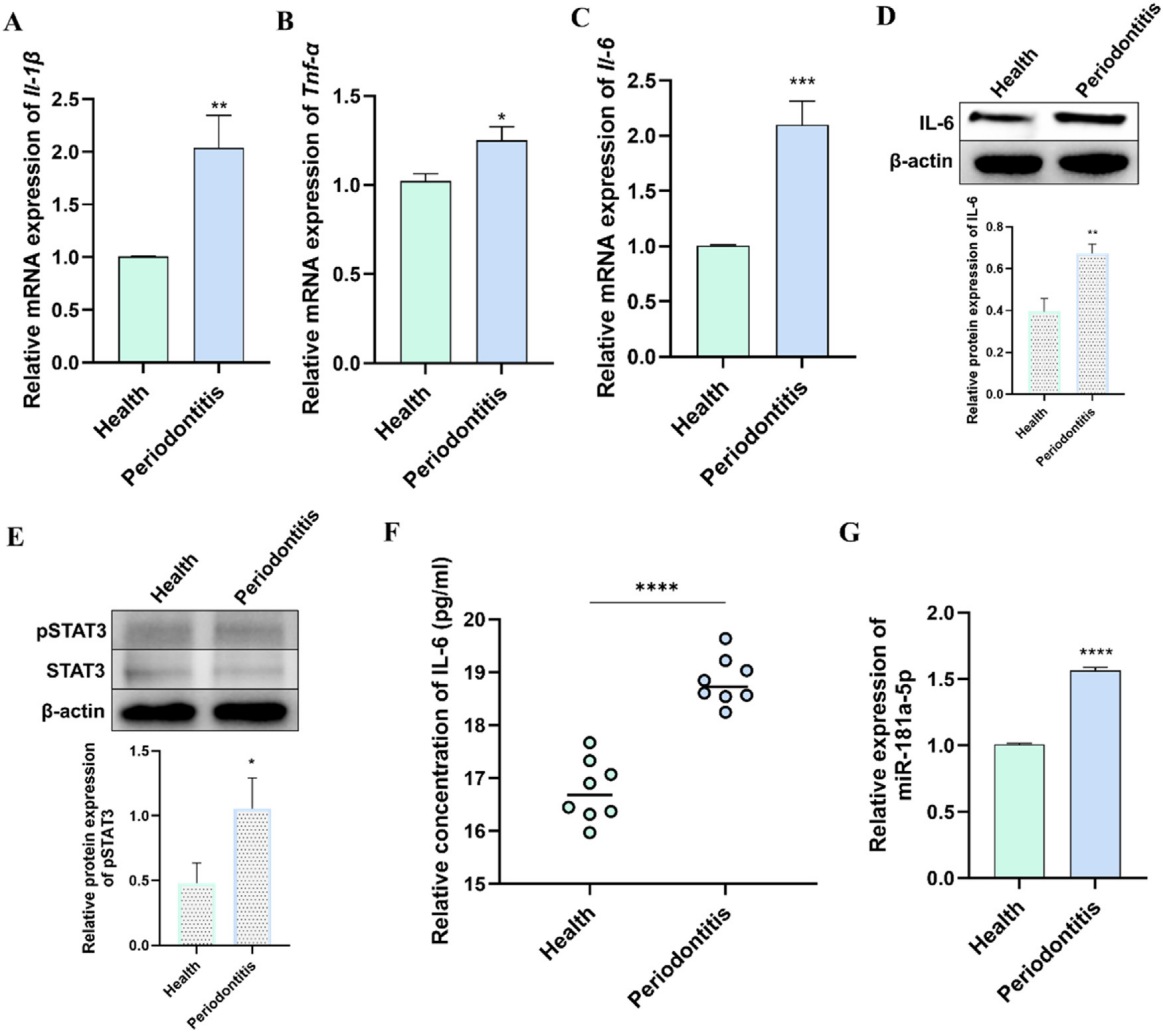
IL-6 and miR-181a-5p were upregulated in periodontitis. (A-C) The expression of *IL-1β, Tnf-α*, and *IL-6* was upregulated in bone marrow–derived mesenchymal stem cells (BMMSCs) from mice with experimental periodontitis. (D, E) The IL-6 upregulation was accompanied by the activity of STAT3/p-STAT3 in periodontitis mBMMSCs. (F) The level of IL-6 was upregulated in the peripheral blood of mice with periodontitis. (G) Compared with the healthy group, the expression of miR-181a-5p was upregulated in periodontitis mBMMSCs. *P < .05, **P < .01, ***P < .001, ****P < .0001.

The cultured mBMMSCs prepared for treatment were able to proliferate and undergo osteogenic differentiation, as well as positively express mesenchymal stem cell surface markers (Figure S2). The expression of *Il-6, Il-1β*, and *Tnf-α* was upregulated in mBMMSCs of periodontitis mice, suggesting the inflammatory situation (Figure 1A-C). In periodontitis mBMMSCs, the upregulation of IL-6 was accompanied by STAT3/p-STAT3 signal activity (Figure 1D, 1E). ELISA found that IL-6 rose in the peripheral blood of periodontitis mice (Figure 1F), while miR-181a-5p was highly expressed in periodontitis-induced mBMMSCs (Figure 1G).

### IL-6 regulated miR-181a-5p expression by STAT3 in mBMMSCs

**Fig. 2:**
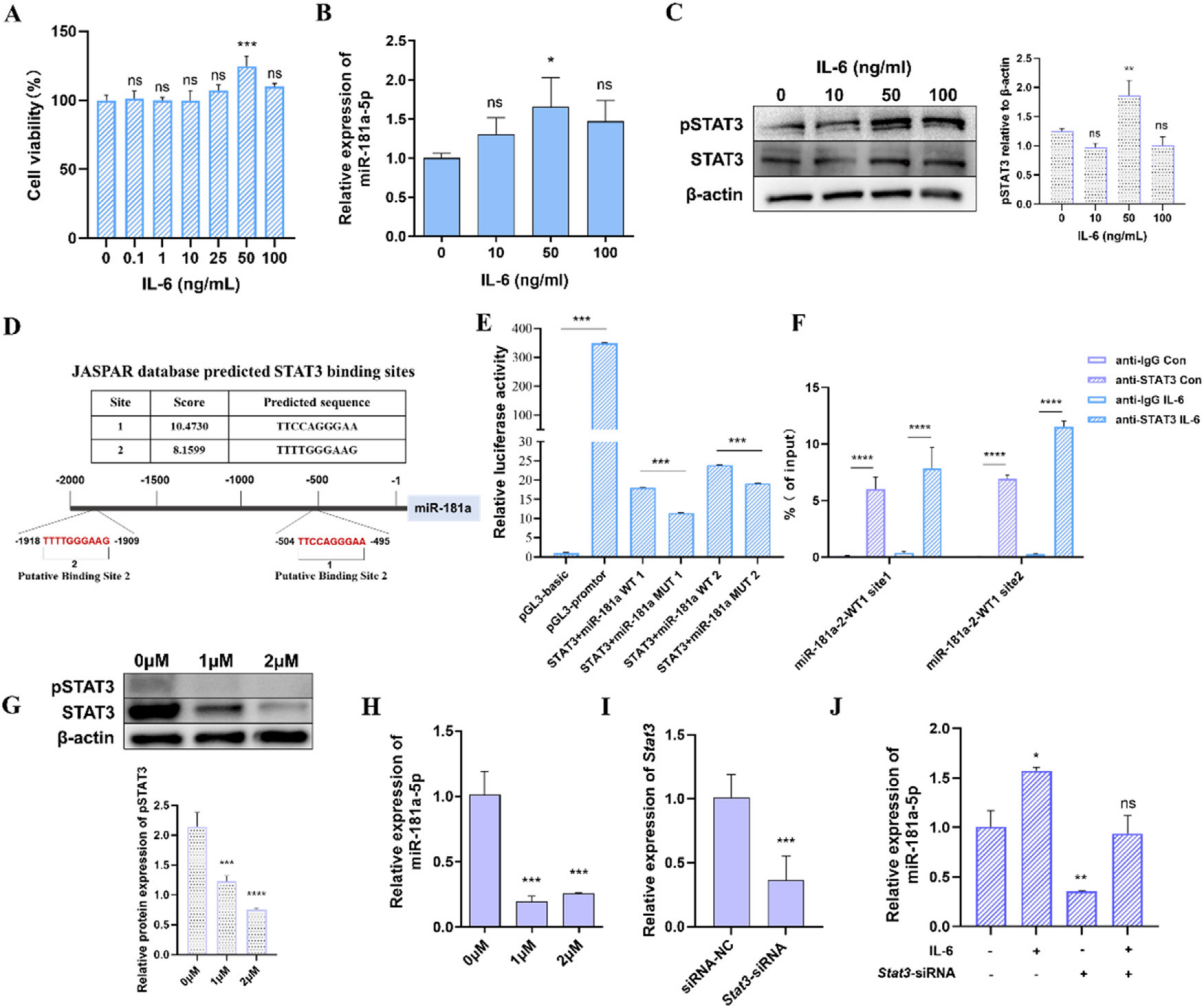
IL-6 regulated miR-181a-5 expression by STAT3 in bone marrow–derived mesenchymal stem cells (BMMSCs). (A) CCK-8 assay of mBMMSCs after treatment with different concentrations of IL-6; 50 ng/mL IL-6 significantly promoted mBMMSC proliferation. (B) A total of 50 ng/mL IL-6 effectively upregulated the expression of miR-181a-5p in mBMMSCs. (C) STAT3 and p-STAT3 levels in mBMMSCs after IL-6 treatment. (D) JASPAR database-predicted binding sites of STAT3 in the miR-181a-5p promoter regions. (E, F) The dual luciferase reporter gene system and chromatin immunoprecipitation–quantitative polymerase chain reaction proved the binding sites of STAT3 on miR-181a-5p promoter regions. (G-I) STAT3 inhibitor and *Stat3*–small interfering RNA (siRNA) significantly inhibited the levels of p-STAT3 in mBMMSCs and downregulated miR-181a-5p. (J) The effects of IL-6 on upregulating miR-181a-5p were reversed by *Stat3*-siRNA. *P < .05, **P < .01, ***P < .001, ****P < .0001; ns, not significant.

Stimulation with different concentrations of IL-6 recombinant protein for 24 hours promoted mBMMSC proliferation and upregulated miR-181a-5p levels. Among these concentrations, 50 ng/mL IL-6 had the most significant effect (Figure 2A, B). Moreover, IL-6 upregulated the level of p-STAT3 and activated the STAT3 pathway (Figure 2C). Analysis of the JASPAR database predicted that STAT3 would bind to the miR-181a-5p promoter regions. Two binding sites with the highest scores were selected and designed for validation of a dual luciferase reporter gene system. Mutation of the predicted STAT3 binding sites on the promoter regions of miR-181a-5p led to a decrease in dual luciferase activity, suggesting that STAT3 could bind to the miR-181a-5p promoter region at site 1 and site 2 (Figure 2D, E). Furthermore, the ChIP-qPCR verified the effects of IL-6 on regulating miR-181a-5p through STAT3. A total of 50 ng/mL IL-6 upregulated the enrichment levels of STAT3 on binding site 1 and site 2 of the promoter regions (Figure S3A), suggesting that IL-6 promoted transcriptional activity by enriching STAT3 levels on the miR-181a-5p promoter regions, thereby upregulating miR-181a-5p (Figure 2F).

STAT3 inhibitor (Figure 2G, H) and *Stat3*-siRNA (Figure 2I, J) transfection downregulated STAT3 transcription and expression levels in mBMMSCs, as well as miR-181a-5p expression. The effects of IL-6 on miR-181a-5p expression could be reversed by STAT3 inhibition (Figure 2J).

### mBMMSCs influenced the Th17/Treg balance via IL-6/STAT3/miR-181a-5p in periodontitis

**Fig. 3:**
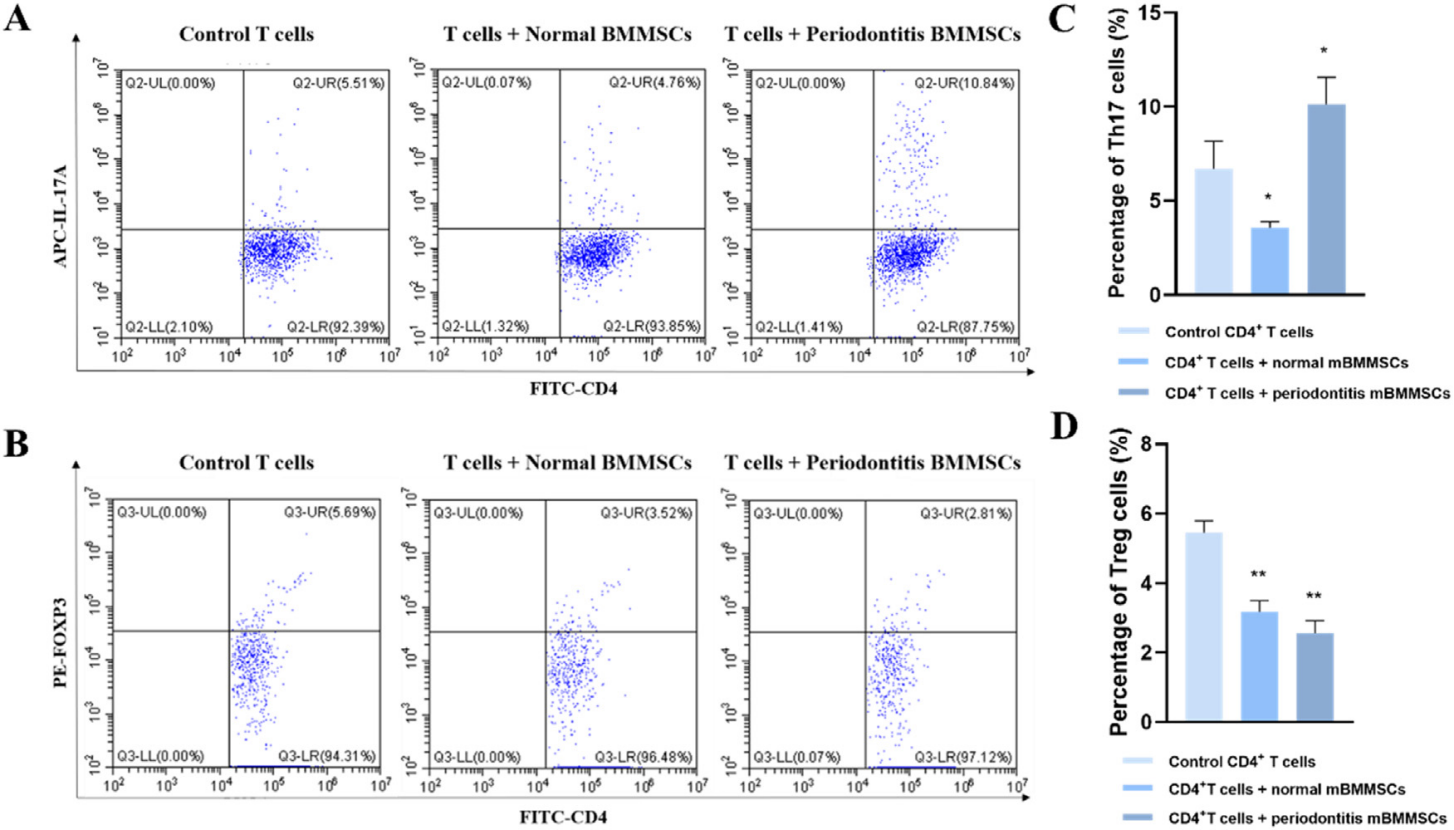
Periodontitis bone marrow–derived mesenchymal stem cells (BMMSCs) influenced the T helper 17 (Th17) cell/regulatory T cell (Treg) balance via IL-6. (A-D) Flow cytometry analysed Tregs and Th17 cells. The CD4^+^ T cells tended to differentiate into Th17 cells after coculturing with mBMMSCs from mice with experimental periodontitis. *P < .05, **P < .01.

**Fig. 4:**
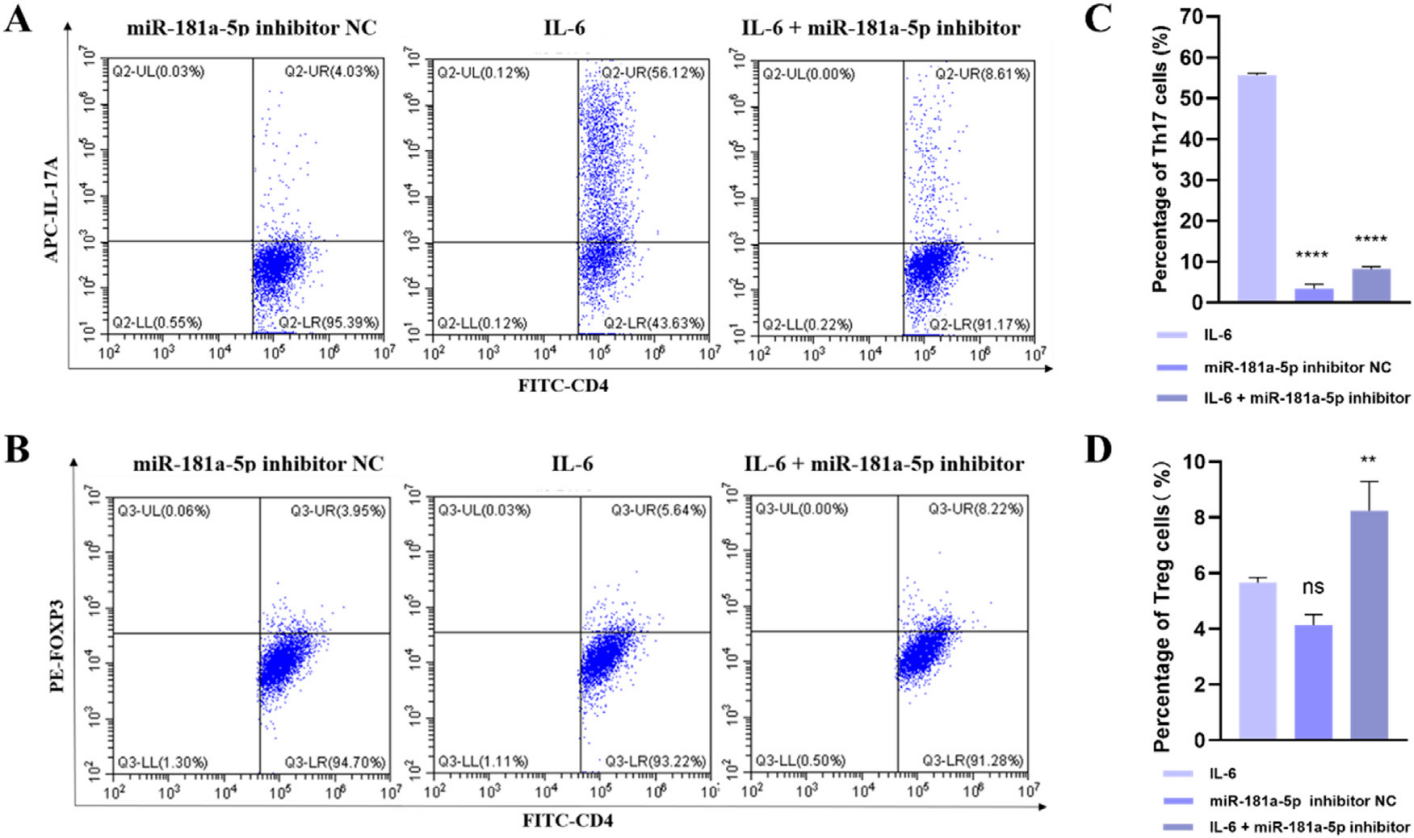
Periodontitis bone marrow–derived mesenchymal stem cells (BMMSCs) influenced the T helper 17 (Th17) cell/regulatory T cell (Treg) balance via miR-181a-5p. (A-D) Flow cytometry analysed Tregs and Th17 cells after CD4^+^ T cells were cocultured with preconditioned mBMMSCs. IL-6–stimulated mBMMSCs promoted Th17 cell differentiation and inhibited Treg differentiation. Functional inhibition of miR-181a-5p alleviated the Th17/Treg imbalance induced by IL-6. **P < .01, ****P < .0001; ns, not significant.

CD4^+^ T cells tended to differentiate into Th17 cells after coculturing with periodontitis mBMMSCs, inducing Th17/Treg imbalance (Figure 3A-D). IL-6–stimulated mBMMSCs promoted Th17 cell differentiation and inhibited Treg differentiation, leading to Th17/Treg imbalance. Functional inhibition of miR-181a-5p alleviated the Th17/Treg immune imbalance triggered by IL-6 stimulation (Figure 4A-D, Figure S3B).

**Fig. 5:**
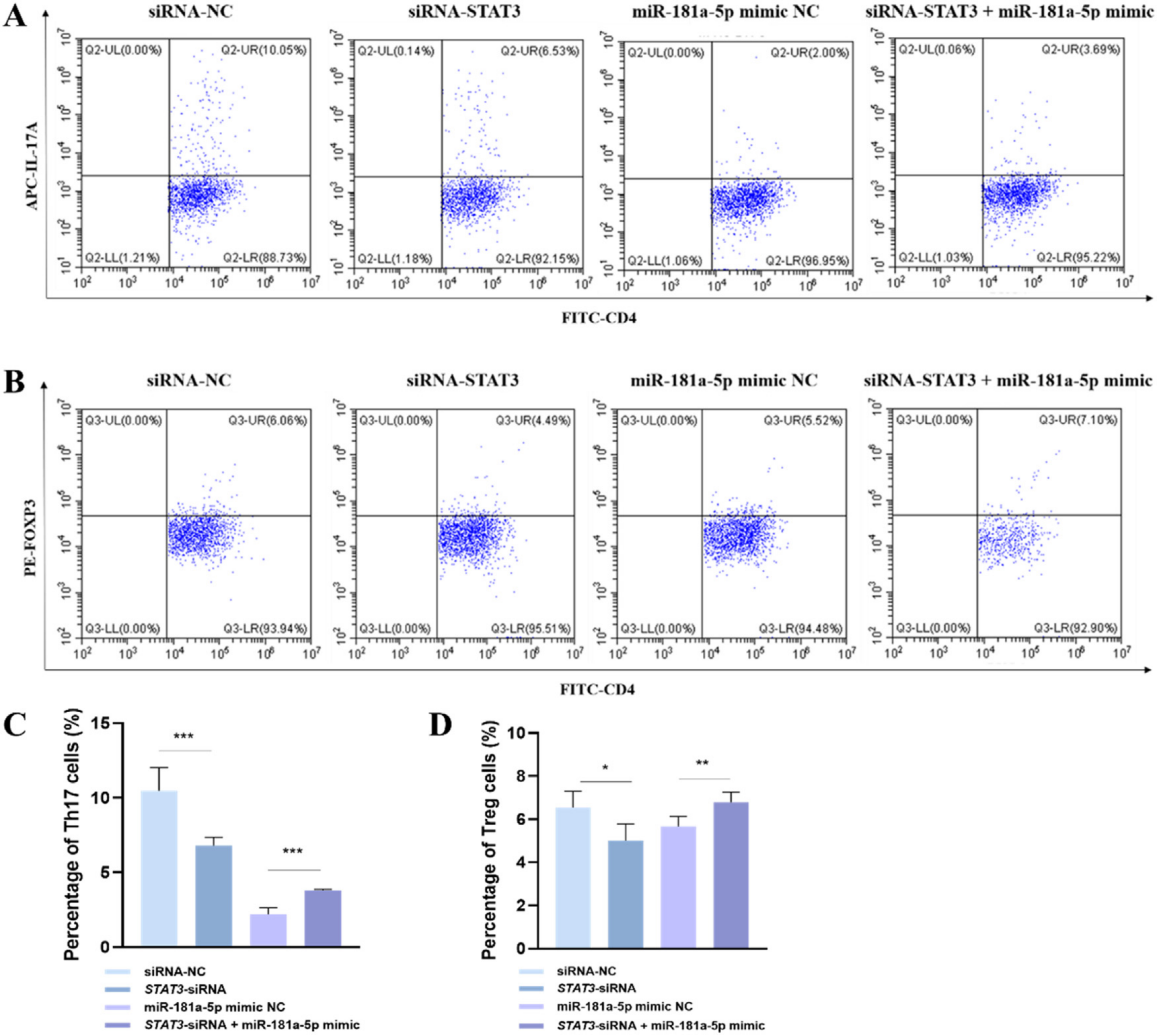
Bone marrow–derived mesenchymal stem cells (BMMSCs) influenced the T helper 17 (Th17) cell/regulatory T cell (Treg) balance via IL-6/STAT3/miR-181a-5p. (A-D) Flow cytometry analysed Th17 cells/Tregs after CD4^+^ T cells were cocultured with preconditioned mBMMSCs. Upregulating the expression of miR-181a-5p in mBMMSCs could partly alleviate the Th17/Treg imbalance induced by Stat3 inhibition. *P < .05, **P < .01, ***P < .001.

Downregulation of *Stat3* in mBMMSCs inhibited Th17 and Treg cell differentiation. Functionally upregulated miR-181a-5p effects on mBMMSCs could partly alleviate Stat3 inhibition-induced Th17/Treg imbalance (Figure 5A-D, Figure S3C).

### miR-181a-5p inhibitor-pretreated mBMMSCs promoted periodontal tissue regeneration in experimental periodontitis

**Fig. 6:**
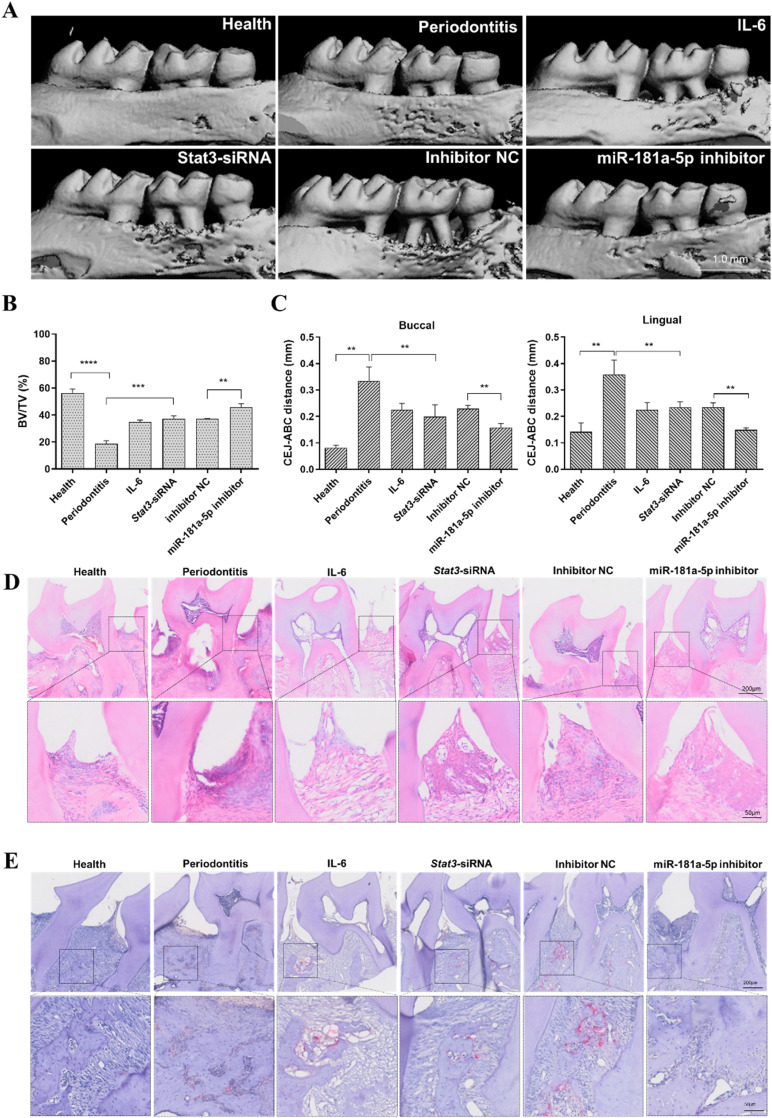
miR-181a-5p inhibitor pretreated bone marrow–derived mesenchymal stem cells (BMMSCs) promoted periodontal tissue regeneration in experimental periodontitis. (A) Micro–computed tomography images of the maxillae of mice. Compared with the periodontitis group, the treatment group with mBMMSCs preconditioned with a miR-181a-5p inhibitor showed alveolar bone recovery. (B, C) Analysis of bone volume/total volume (BV/TV) and the cementoenamel junction–alveolar bone crest (CEJ-ABC) distances of the buccal and palatal surfaces. The ratio of BV/TV upregulated significantly in the miR-181a-5p inhibitor preconditioned group, while the CEJ-ABC distance in the miR-181a-5p inhibitor group was significantly reduced. (D, E) Haematoxylin and eosin and tartrate-resistant acid phosphatase analysis of periodontal tissues. After injection of mBMMSCs pretreated with IL-6, periodontal tissues were destroyed and infiltrated by inflammatory cells. The periodontal tissues of the miR-181a-5p inhibitor treatment group showed obvious recovery, and osteoclasts and bone resorption reduced significantly. **P < .01, ***P < .001, ****P < .0001.

Micro-CT analysis showed that compared with periodontitis mice, treatment with different pretreated mBMMSCs exerted differential effects on alveolar bone recovery. The ratio of BV/TV was upregulated significantly in miR-181a-5p inhibitor-treated groups, while CEJ-ABC in the miR-181a-5p inhibitor group was reduced with statistically significant differences (Figure 6A-C). The results of histologic analysis showed that the periodontal tissues of mice injected with mBMMSCs pretreated with IL-6 were destroyed and infiltrated by inflammatory cells. The periodontal tissues of the miR-181a-5p inhibitor treatment group showed obvious recovery, as well as a decrease in the number of osteoclasts and a significant reduction in bone resorption (Figure 6D, E).

**Fig. 7:**
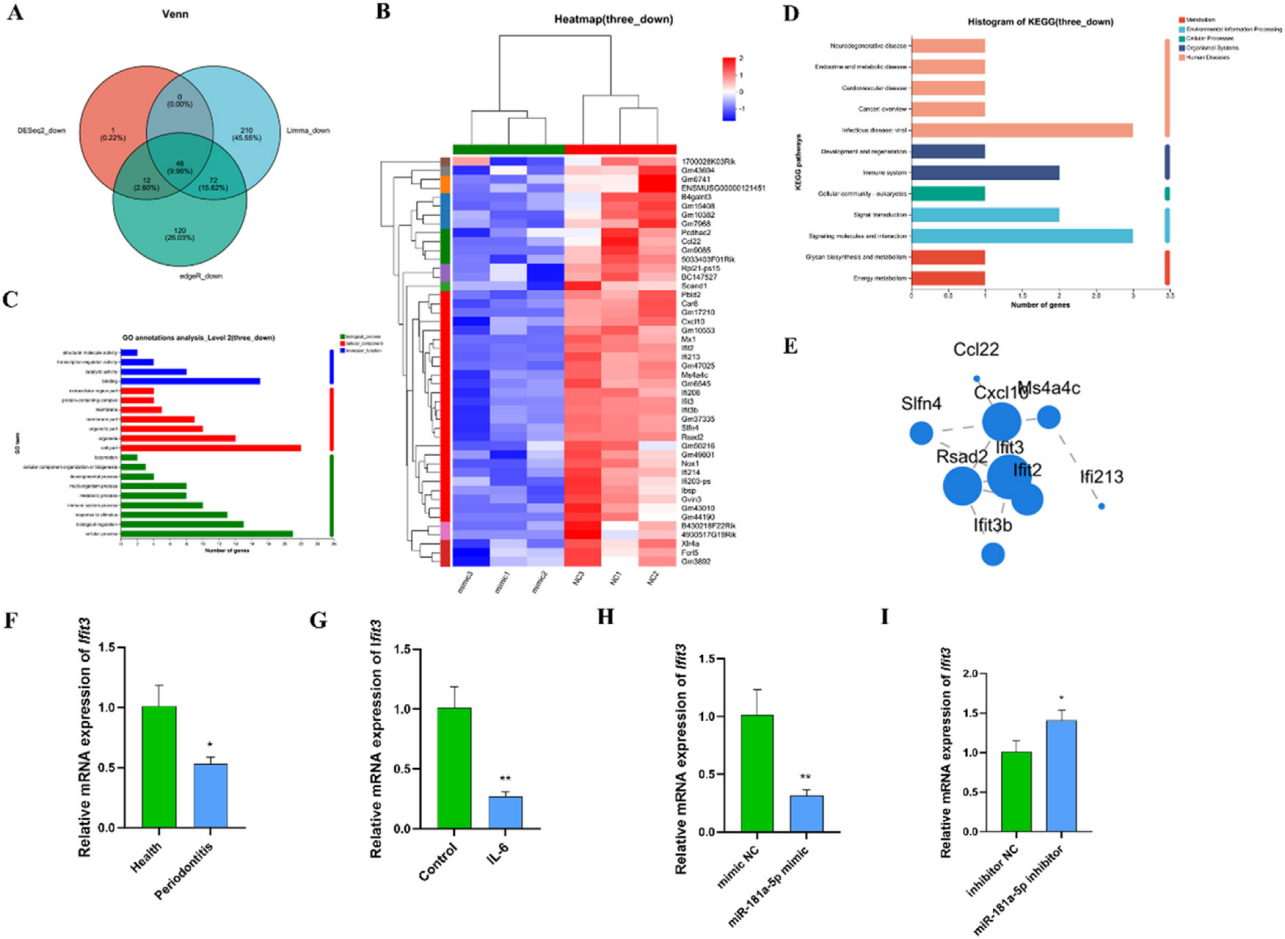
miR-181a-5p was predicted to target *Ifit3*. (A, B) Venn plot and visualisation heatmap of RNA sequencing (RNA-seq results). (C, D) Gene Ontology and Kyoto Encyclopedia of Genes and Genomes analysis. (E) The top 9 downregulated genes of RNA-seq. (F, G) The expression of *Ifit3* in periodontitis and IL-6–pretreated bone marrow–derived mesenchymal stem cells was downregulated. (H, I) The expression of *Ifit3* was inhibited after miR-181a-5p was functionally upregulated. Conversely, miR-181a-5p inhibition promoted *Ifit3* expression. *P < .05, **P < .01.

To investigate the miR-181a-5p target genes and functions in periodontal regeneration, mBMMSCs were treated with miR-181a-5p mimic, and cell transcriptome RNA-seq was performed. Three statistical analyses of DESeq2, Limma, and edgeR were used to examine the sequencing results and generate a Venn plot and a visualisation heatmap. As a result, there were 46 genes significantly downregulated (Figure 7A, B). The Gene Ontology analysis showed that differential genes were classified and annotated into 3 main kinds: “molecular function,” “cellular component,” and “biological process” (Figure 7C). The Kyoto Encyclopedia of Genes and Genomes analysis showed that the differentially expressed genes were enriched in the contexts of “organismal systems, metabolism, human diseases, and cellular processes” (Figure 7D).

The STRING database was used to analyse these downregulated genes and screen out the top 9 differentially expressed genes (Figure 7E), including *Ccl22, Slfn4, Cxcl1, Ms4a4c, Rsad2, Ifit3, Ifit2, Ifi213*, and *Ifit3b*. The TargetScan database was used to predict the potential target gene and binding site of miR-181a-5p. *Ifit3* was the only gene that was predicted to be targeted by miR-181a-5p. According to qRT-PCR results, the expression levels of *Ifit3* in periodontitis mBMMSCs and IL-6 pretreated mBMMSCs were also downregulated. Furthermore, *Ifit3* expression was inhibited after miR-181a-5p was functionally upregulated. Conversely, miR-181a-5p inhibition promoted *Ifit3* expression (Figure 7F-I).

## Discussion

Periodontal tissue regeneration and repair are always a focus of periodontitis-related research.^24^^,^^25^ BMMSCs not only have functions of mesenchymal stem cells, such as self-renewal, proliferation, and multipotential differentiation properties, but also have the advantage of being an abundant source.^26^^,^^27^ In recent years, BMMSCs have been regarded as promising candidates for regenerative therapy of periodontitis because of their regulatory effects on immune cells.^9^ The mBMMSCs isolated and cultured in the present study could grow like a colony, with strong proliferation and osteogenic differentiation ability, as well as have positive expression of mesenchymal stem cell surface antigen markers CD90 and CD105, coinciding with previous reports.^28^ Studies have found that the mBMMSCs locally transplanted could be recruited to periodontal tissue defects and exert immune-regulatory effects by inducing T helper cell apoptosis through FasL, which helps periodontal tissue regeneration.^14^^,^^29^, ^30^, ^31^ The immune-regulatory function of mesenchymal stem cells is achieved by intercellular contact, cellular factors, and extracellular vesicles, including small molecules such as miRNAs.^32^, ^33^, ^34^ As a kind of biological molecule agent, microRNAs have a certain effect on immune regulation, with previous studies proving that miR-181a-5p inhibited CD4^+^ T lymphocyte apoptosis by FasL expression in mBMMSCs.^23^^,^^35^ The results of this study further verify the immunomodulatory function of miR-181a-5p, which has been validated in an experimental periodontitis model. It deepens understanding of osteoimmunologic regulation in periodontal inflammatory damage and elaborates the interactive mechanism among inflammatory factors, microRNAs, and immune cell homeostasis.

The elevation of IL-6 directly participates in the inflammatory cascade of periodontitis, inducing periodontal tissue destruction.^36^^,^^37^ The present research studied experimental periodontitis in mice and found that IL-6 was upregulated in both peripheral blood and mBMMSCs. To date, IL-6 has been shown to regulate the expression of certain miRNAs in several inflammatory diseases by activating the STAT3 pathway.^38^ IL-6 promoted nuclear translocation of phosphorylated STAT3 and combined with miR-135b promoter regions.^39^ In chronic lymphocytic leukaemia, IL-6 upregulated miR-155 expression by activating Stat3, and siRNA-*Stat3* inhibited the expression level of miR-155.^40^ IL-6–induced phosphorylation of STAT3 inhibited the transcription of miR-34a, further mediating invasion of rectal cancer.^20^^,^^41^ In the present study, a series of experiments were performed to explore and verify the immune-regulatory ability of IL-6 on mBMMSCs and miR-181a-5p in the inflammatory microenvironment of periodontitis. As a result, IL-6 can upregulate miR-181a-5p expression by activating STAT3. The IL-6/STAT3/miR-181a-5p axis was involved in the resolution of periodontitis.

As a classical signalling pathway, the IL-6/JAK/STAT3 axis is closely related to the occurrence and development of inflammation.^42^^,^^43^ Specific inhibition of the IL-6 receptor (IL-6R) could alleviate inflammatory bone resorption in experimental periodontitis.^44^ As a biological factor related to cell proliferation, cell differentiation, and inflammatory signalling molecules, the status of STAT3 is closely associated with the inflammatory factor IL-6 and its upregulation.^45^^,^^46^ In T cells and epithelial cells of periodontitis, STAT3 activation was increased and necessary for producing IL-17A.^47^ Mut-*Stat3* mice showed less alveolar bone resorption than periodontitis mice, suggesting STAT3 was a pathogenic factor in inflammatory alveolar bone loss.^48^ Studies have proven that LncRNA-SPIRE1 competitively absorbs miR-181-5p and further regulates JAK/STAT3, thereby modulating the immune effects of mBMMSCs on the Th17/Treg balance.^49^ The present research found that IL-6–preconditioned mBMMSCs promote CD4^+^ T cells to differentiate into Th17 cells, inducing Th17/Treg imbalance. However, the miR-181a-5p inhibitor can reverse the Th17/Treg imbalance caused by the above mechanism. The STAT3 inhibition of mBMMSCs suppressed Th17 cell differentiation, and these effects were reversed by the miR-181a-5p mimic. All these suggested that IL-6 regulated miR-181a-5p in mBMMSCs via STAT3, thereby influencing the immune-regulatory ability of mBMMSCs. From a clinical perspective, this regulatory axis provides novel molecular targets for immune intervention therapy in periodontitis. Targeted regulation of miR-181a-5p may help restore local immune homeostasis, alleviate periodontal inflammation, and facilitate periodontal tissue repair.

For our experimental periodontitis mice, miR-181a-5p inhibition of mBMMSCs effectively promoted periodontal tissue repair. The RNA-seq results and predictions, along with the database, suggested that *Ifit3* may be targeted by miR-181a-5p. IFIT3 is associated with antiviral innate immunity, affecting immune response by regulating the degree of immune cell differentiation.^50^^,^^51^ IFIT3 has been proven to be targeted by miR-4756 and takes part in the immune regulation of cancer pathogenesis.^52^ Based on the evidence above, *Ifit3* may be a meaningful downstream target gene involved in immune regulation of miR-181a-5p in BMMSCs, laying the foundation for deeper research related to miR-181a-5p functions on immune regulation. Nevertheless, our study still has certain limitations. The mechanistic validation of downstream target Ifit3 is preliminary, and in vivo functional rescue assays are absent. In addition, evidence from clinical human samples is insufficient, which restricts the direct clinical application of our conclusions. Further studies are required for in-depth mechanism verification and translational exploration.

For future research, further in vivo rescue experiments and clinical sample detection will be conducted to verify the complete regulatory pathway. Moreover, deeper mechanistic exploration and targeted intervention studies will be carried out to promote the clinical transformation of the above molecular targets for periodontal immunotherapy and tissue regeneration.

## Conclusions

The present study clarified the mechanistic findings of the IL-6/STAT3/miR-181a-5p axis in BMMSC-mediated periodontal regeneration. IL-6 may promote STAT3 activation to modulate miR-181a-5p expression. The subsequent targeted regulation of Ifit3 could partially restore Th17/Treg immune imbalance, which might ultimately alleviate inflammatory alveolar bone destruction in periodontitis. These results extend the regulatory mechanism of the classical IL-6/STAT3 pathway and deepen the understanding of BMMSC immunomodulation in periodontal immune disorder. The identified molecular axis lays a theoretical foundation for developing novel immunomodulatory strategies and highlights the therapeutic potential of miR-181a-5p–targeted intervention combined with BMMSC therapy in periodontal regenerative medicine. However, several limitations in the present study, as well as the long-term biosafety and systemic effects of stem cell treatment, have not been fully evaluated. Further studies are still required to deeply clarify the underlying role of Ifit3 in this regulatory mechanism and further explore the feasibility of clinical application.

## Author Contributions

Deqin Yang and Si Wu conceived and designed these experiments. Si Wu and Jie Wang performed these experiments. Jie Wang analysed and interpreted the data. Si Wu wrote the manuscript. Deqin Yang revised the manuscript. All authors read and approved the final manuscript.

## Conflict of interest

The authors declare that they have no known competing financial interests or personal relationships that could have appeared to influence the work reported in this paper.

## Appendix Supplementary materials

Image, application 1

## Footnotes

Supplementary material associated with this article can be found in the online version at doi:10.1016/j.identj.2026.109675.
