# Supplementary material for: IL-6/STAT3–Mediated miR-181a-5p in Bone Marrow–Derived Mesenchymal Stem Cells Regulates Th17/Treg Balance in Experimental Periodontitis
Source: Int Dent J. 2026 Jun 11;76(4):109675. doi: 10.1016/j.identj.2026.109675 (PMC13276558; doi:10.1016/j.identj.2026.109675)
Supplement: Supplementary file 1 [file mmc1.docx]

**Supplementary Materials**


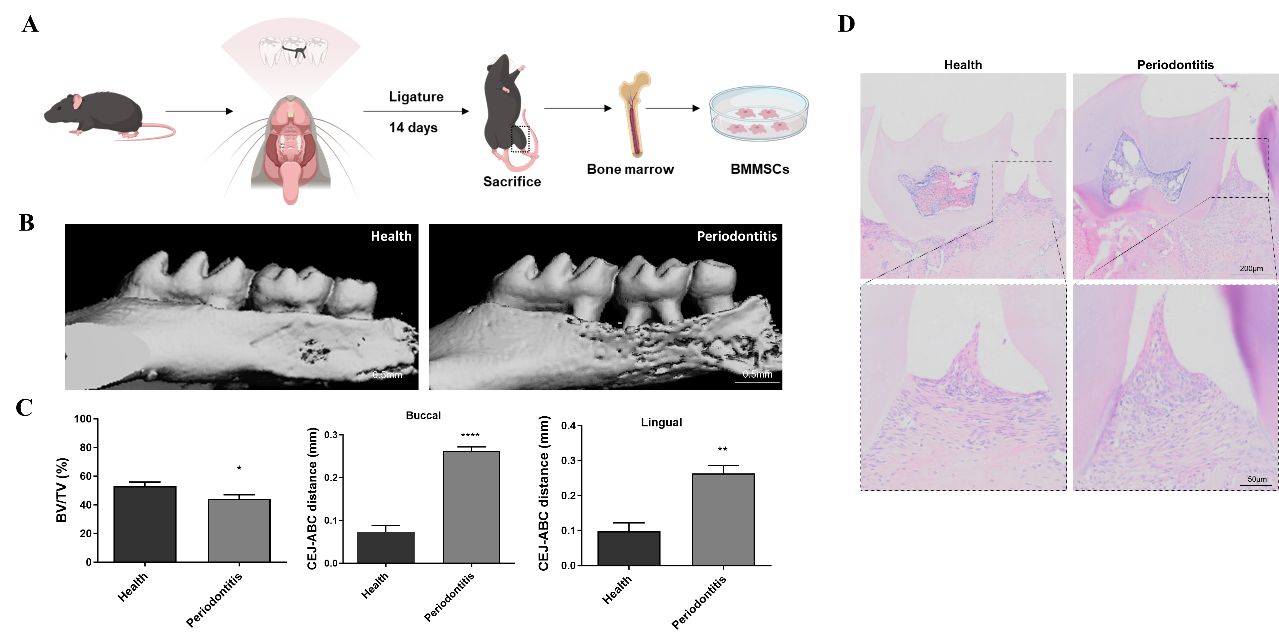


**Supplementary Figure 1** (A) The periodontitis was established in the maxillary second molar of mice by silk ligation method. (B) MicroCT images of maxillary of mice. (C) Analysis of BV/TV and the CEJ-ABC distance of buccal and palates. (D) Histological analyses of the periodontal tissues by H&E staining. (**p* < 0.05, ***p* < 0.01, *****p* < 0.0001)


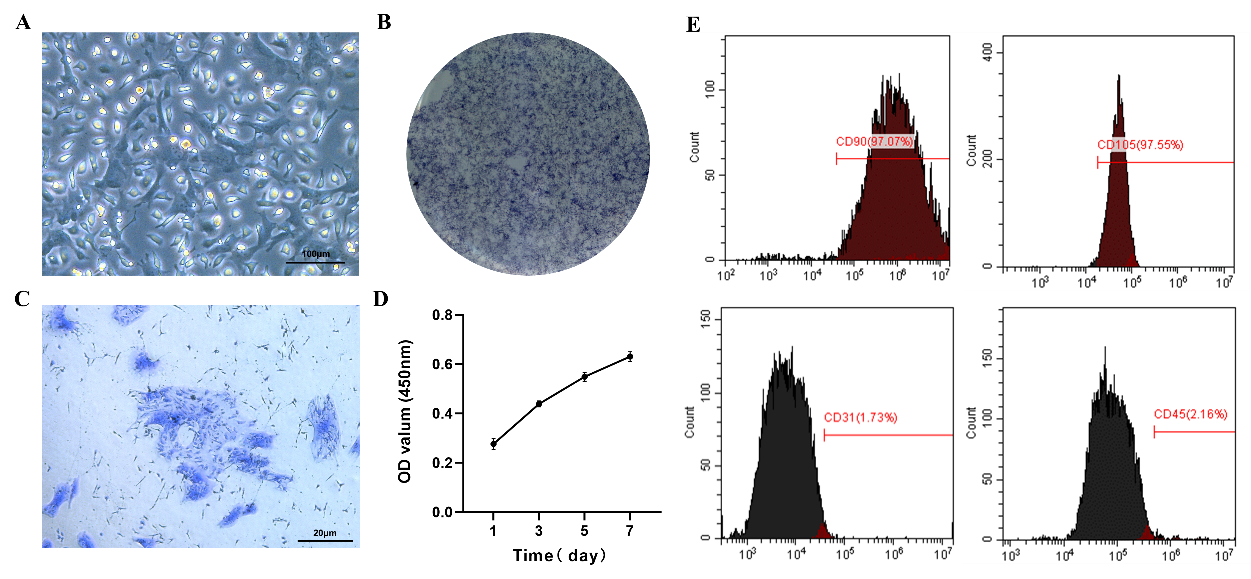


**Supplementary Figure 2** (A) The appearances of cultured mBMMSCs. (B) ALP staining of mBMMSCs after osteogenic induction. (C) Crystal violet staining of mBMMSCs. (D) CCK8 analysis of mBMMSCs. (E) Flow cytometry analysis of mBMMSCs.


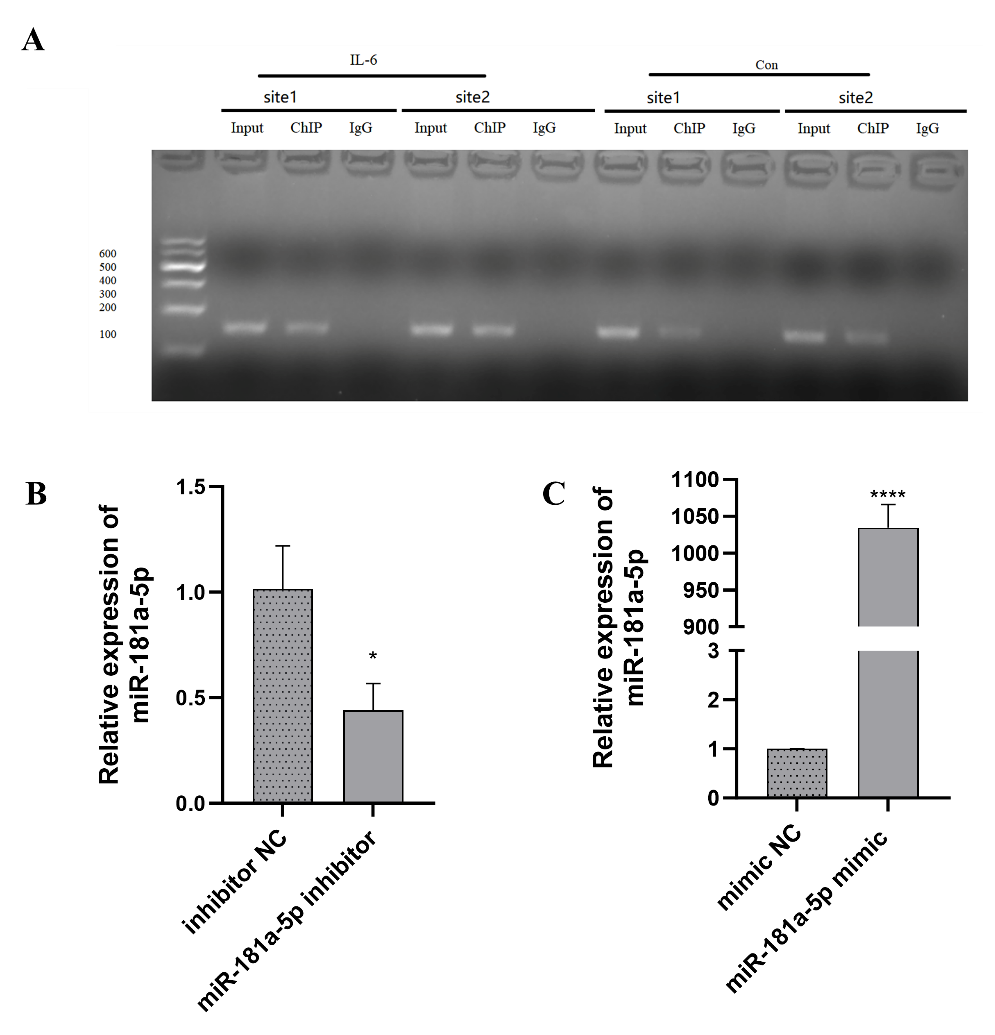


**Supplementary Figure 3** (A) The enrichment of STAT3 on promoter regions of miR-181a-5p. (B, C) Transfection efficiency of miR-181a-5p mimic and miR-181a-5p inhibitor. (**p* < 0.05, *****p* < 0.0001)
